# From mental strain to gut pain: A brain‐gut pathway transducing psychological stress to intestinal inflammation

**DOI:** 10.1002/ctm2.1458

**Published:** 2023-10-27

**Authors:** Kai Markus Schneider, Niklas Blank, Christoph A. Thaiss

**Affiliations:** ^1^ Microbiology Department, Institute for Immunology, and Institute for Diabetes Obesity and Metabolism, Perelman School of Medicine, University of Pennsylvania Philadelphia Pennsylvania USA; ^2^ Department of Medicine III University Hospital RWTH Aachen Aachen Germany

## Abstract

Psychological stress can trigger inflammatory bowel disease (IBD) flares, but the molecular mechanisms have remained unclear. We recently discovered an unexpected function of the enteric nervous system as a relay between stress signals from the brain and intestinal inflammation. Our findings highlight targeting stress‐induced signaling networks as a possible new pillar in the clinical management of IBD.

## 1 BRIDGING MIND AND GUT

The interconnectedness of our mental state with our physical well‐being is a subject that has fascinated both scientists and philosophers for centuries. Recent developments in biomedical technologies enable new perspectives on this long‐standing question and have started to unveil the multitude of communication routes between the nervous system and periphery.

The connection between brain and gut is particularly evident in inflammatory bowel disease (IBD), which is an umbrella term that describes disorders involving chronic inflammation in the gastrointestinal tract and manifests in two main forms: ulcerative colitis and Crohn's disease.^1^ Approximately three million adults in the U.S. experience severe pain, bleeding, weight loss, and frequent hospitalizations due to IBD. Treatment typically includes immunosuppressants like steroids or biologicals. However, even when managed properly, IBD can lead to sporadic flare‐ups, and pinpointing their exact causes has been challenging. Previous epidemiological studies have provided evidence that stressful life events can intensify IBD flares. However, the underlying mechanisms and brain‐to‐gut pathways transducing psychological stress to intestinal inflammation during IBD flare‐ups are incompletely understood.

## 2 STRESS IN IBD: THE CHICKEN OR THE EGG?

Several studies have explored the psychological impact of IBD. A meta‐analysis found a high prevalence of anxiety and depression among IBD patients.^2^ Psychological distress may stem from the unpredictability of disease flare‐ups, the need for chronic medication or even surgery, social stigma, dietary restrictions, and an overall reduction in the quality of life.^3^ The bidirectional relationship between stress and IBD, however, makes it challenging to delineate cause and consequence in epidemiological studies.

We set out to address this conundrum by establishing an experimental model that could mimic the effects of psychological stress on IBD. Using the dextran sodium sulfate (DSS) and IL‐10 deficiency models of gut inflammation, we simulated psychological stress by physically restraining mice or exposing them to dominant “aggressor” mice, methods previously employed in stress research.^4^ Notably, both stress paradigms exacerbated colitis in mice.^5^

## 3 HOW DOES STRESS EXACERBATE COLITIS?

The organism's response to psychological stress has traditionally been understood to involve activation of two key pathways: the sympathetic nervous system and the hypothalamic‐pituitary‐adrenal axis. We verified stress‐induced responses in both sympathetic signaling and an increase in corticosterone secreted by the adrenal gland. Interestingly, only targeted inhibition of corticosterone signaling provided protection from the deleterious impact of psychological stress on gut inflammation.

**FIGURE 1:**
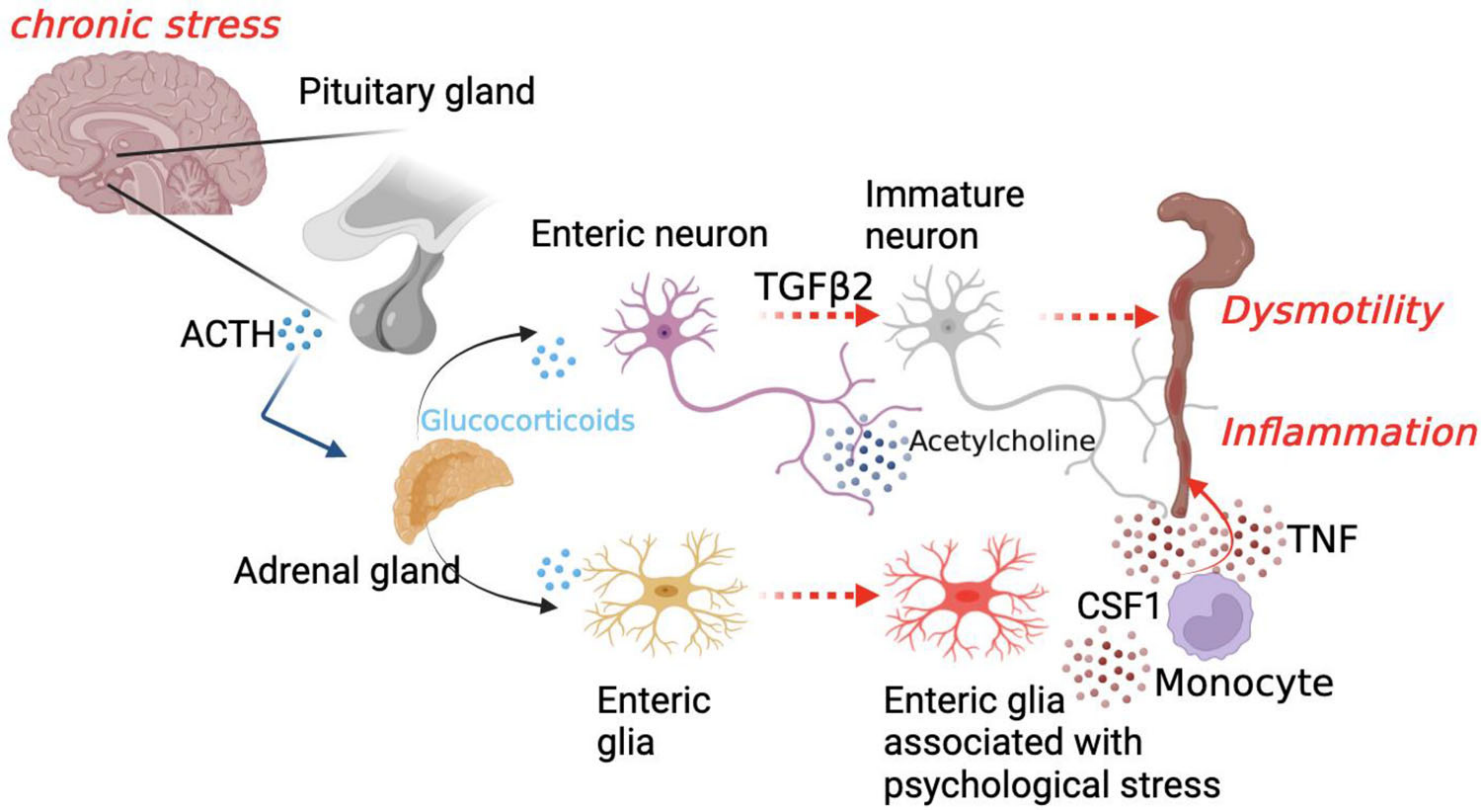
Schematic of the brain‐to‐gut circuit by which psychological stress exacerbates intestinal inflammation. ACTH, Adrenocorticotropic‐Hormon, CSF1, Colony stimulating Factor 1, TGFß2, Transforming growth factor beta 2.

Using a combination of flow cytometry and single‐cell transcriptome analyses, we identified tumor necrosis factor (TNF)‐expressing monocytes as key mediators of stress‐induced colitis exacerbation (Figure 1). However, contrary to our expectations, deletion of the glucocorticoid receptor gene *Nr3c1* from myeloid cells did not result in protection from the adverse effects of stress. Rather, glucocorticoid signaling in the enteric nervous system (ENS) appeared to mediate the impact of chronic psychological stress on intestinal inflammation.^5^

## 4 THE ENTERIC NERVOUS SYSTEM RELAYS PSYCHOLOGICAL STRESS TO INTESTINAL INFLAMMATION

Like brain, the ENS is composed of a tight network of neurons and glial cells.^6^ We therefore studied the transcriptomic landscape of the ENS during chronic stress using single‐nucleus RNA‐sequencing. Interestingly, stress resulted in profound transcriptomic changes in the ENS, specifically the emergence of a cluster of transcriptionally activated inflammatory glial cells, which we termed enteric glia associated with psychological stress. Cell‐cell interaction analyses based on our transcriptomic data revealed that these cells produce CSF1, a cytokine that attracts monocytes. Additionally, we found that stress leads to transcriptional immaturity of enteric neurons via TGFβ2, which resulted in constipation and dysmotility.^5^

## 5 STRESS MANAGEMENT AS A THERAPEUTIC PILLAR IN IBD CARE

We validated our findings from experimental models in several human cohort studies. Psychological stress was associated with higher incidence and increased severity of colitis in the population‐based UK Biobank study, the myIBDcoach real‐world prospective cohort study, as well as a prospective colonoscopy study with biopsies that we had recruited.^5^, ^7^, ^8^ In addition, perceived stress levels in individuals with IBD positively correlated with transcripts indicative of monocyte recruitment, myeloid‐cell driven inflammation, TNF production, as well as expression of *TGFB2*.

Collectively, our study demonstrates that the ENS is significantly influenced by signals originating from the central nervous system (CNS). Specifically, enteric glial cells may play a broader role in communication of the CNS with the ENS. Our study focused on brain‐to‐gut communication, but the existence of ENS‐to‐brain communication routes during psychological stress are well conceivable.

Clinically, our study tackles the paradox surrounding corticosteroids being beneficial in acute treatment but associated with unfavorable outcomes upon chronic treatment. We find that chronically elevated glucocorticoid levels transcriptionally reprogram enteric glial cells, which in turn aggravate colitis. Our data could offer new perspectives on the limitations of prolonged corticosteroid therapy for IBD.

Finally, our study emphasizes the often‐overlooked impact of a patient's psychological state on the efficacy of IBD treatment and suggests that treatments may have variable outcomes depending on an individual patient's mental state. Our results thus call for the integration of psychological evaluation and stress reduction strategies in clinical settings (Figure 1).
